# Air exchanges, climate change, and severe acute respiratory coronavirus virus 2 (SARS-CoV-2): Results from a survey of the Society of Healthcare Epidemiology of America Research Network (SRN)

**DOI:** 10.1017/ash.2021.256

**Published:** 2022-03-11

**Authors:** Jason P. Burnham, Fred Betz, Roger Lautz, Ehsan Mousavi, Richard A. Martinello, Forbes McGain, Jodi D. Sherman

**Affiliations:** 1 Division of Infectious Diseases, Washington University in St Louis School of Medicine, St Louis, Missouri, United States; 2 Affiliated Engineers, Building Performance Practice/Intelligent Buildings Practice, Madison, Wisconsin, United States; 3 Department of Construction Science and Management, College of Architecture, Arts, and Humanities, Clemson University, Clemson, South Carolina, United States; 4 Departments of Internal Medicine and Pediatrics, Yale School of Medicine, New Haven, Connecticut, United States; 5 Division of Quality and Safety, Yale New Haven Health, New Haven, Connecticut, United States; 6 Anaesthesia and Intensive Care, Western Health, Melbourne, Australia; 7 Department of Critical Care, University of Melbourne, Melbourne, Victoria, Australia; 8 School of Public Health, University of Sydney, Sydney, New South Wales, Australia; 9 Department of Anesthesiology, Yale School of Medicine, New Haven, Connecticut, United States; 10 Department of Environmental Health Sciences, Yale School of Public Health, New Haven, Connecticut, United States

**Keywords:** air exchanges, climate change, COVID-19, HVAC, operating rooms

## Abstract

In this cross-sectional survey, we assessed knowledge, attitudes and behaviors regarding operating room air-change rates, climate change, and coronavirus disease 2019 (COVID-19) pandemic implications. Climate change and healthcare pollution were considered problematic. Respondents checked air exchange rates for COVID-19 and ∼25% increased them. Respondents had difficulty completing questions concerning hospital heating, ventilation and air conditioning (HVAC) systems.

Climate change is the greatest public health threat of the 21st century.^1^ Healthcare is a major polluting industry, responsible for 8.5% of US greenhouse gas emissions.^2^ Mitigating healthcare pollution is integral to first doing no harm. Heating, ventilation, and air conditioning (HVAC) accounts for 70%–97% of operating room (OR) energy usage.^3,4^ Healthcare HVAC systems could be safely manipulated to reduce energy use and environmental impact.^5^

The American Society for Health Care Engineering (ASHE) hospital HVAC recommendations for ORs (unchanged during our survey) include (1) minimum efficiency reporting value (MERV) filter ratings of ≥14, (2) positive pressure ORs, and (3) minimum of 20 air changes per hour (4 outdoor air changes per hour minimum).^6^ In a 2019 review of ASHE HVAC recommendations, outdoor air changes per hour and minimum total OR air changes per hour were rated as needing ‘further investigation’ due to little supporting clinical evidence, and other hospital area parameters had little or poor-quality evidence.^7^ Current standards are based on staff comfort, odor control, fire prevention, and infection prevention; however, little evidence correlates air-change rates and outcomes including surgical site infections (SSIs).^7^

We surveyed current OR air-change rate practices, including any set-back transitions (ie, decreasing air changes during off hours), as well as attitudes about climate change, and OR change rates in light of the COVID-19 pandemic.

## Methods

The Washington University School of Medicine Institutional Review Board approved the survey, and it was pilot tested with 5 experts. The 36-item anonymous survey was distributed to the American Hospital Association (AHA) by nondedicated e-mail and the Society for Healthcare Epidemiology of America Research Network (SRN) by dedicated e-mail from October 2020 to February 2021. Within the SRN, 67 US institutions (1 recipient per hospital) received 4 notifications. Data were collected using Qualtrics software (Seattle, WA). Descriptive statistical analyses were performed using SPSS version 27 software (IBM, Armonk, NY).

## Results

**Fig. 1.**
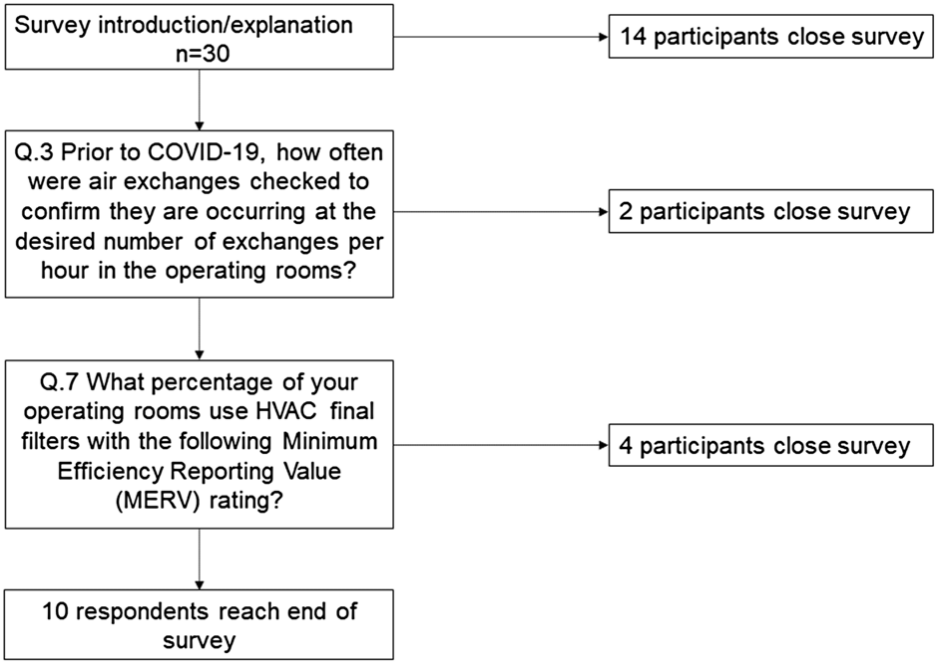
Flow diagram of participants’ survey completion.

The AHA survey recipients had response rates <1% and were excluded from analysis. Of 67 SRN participants, 30 (45%) opened the survey. Of these 30 participants, 10 (15%) completed the survey, and 20 (30%) partially completed it (Fig. 1).

Among respondents, 7 (70%) were male, 8 (80%) were white, 7 (70%) were aged >50 years, 8 (80%) were hospital epidemiologists, and 2 (20%) were infection preventionists. Six respondents were from academic hospitals, 3 were from a community-based hospital, and 1 was from a Veterans Affairs hospital. The hospiitals of 80% of respondents had >250 beds.

### Climate

Overall, 10 (100%) respondents agreed or strongly agreed that “pollution caused by the healthcare industry is important to minimize” and “I am concerned about climate change” (Table 1). Regarding the statement “There is no limit to the amount of resources we should use to prevent even one patient from developing a hospital-acquired infection,” 1 (10%) agreed or strongly agreed, 3 (30%) were neutral, and 6 (60%) disagreed or strongly disagreed.

**Table 1.** Statement and Responses Regarding Climate Change, Energy, and Infections

**What percentage of your operating rooms use the following minimum air exchanges per hour during surgeries? (N = 16), mean %**						
≤12	13–16	17–20	>20			
air exchanges per hour	air exchanges per hour	air exchanges per hour	air exchanges per hour			
6.8	23.4	32.2	37.5			
**Do you decrease the air exchange rates in your operating rooms during off hours (eg, nights/weekends)? (N = 16), no. (%)**						
Yes	No					
2 (12.5)	14 (87.5)					
**What is the air exchange rate decreased to during off hours (air exchanges/hour)? (N = 2)**						
13–16 air exchanges per hour		>20 air exchanges per hour				
1		1				
**What percentage of operating rooms have their air exchange rate decreased during off hours? (N = 2), mean %**						
Decreased to ≤12 air exchanges per hour	Decreased to 13–16 air exchanges per hour	Decreased to 17–20 air exchanges per hour	Decreased to >20 air exchanges per hour	No change in air exchanges per hour		
0	15	0	0	85		
**Prior to COVID-19, how often were air exchanges checked to confirm they are occurring at the desired number of exchanges per hour in the operating rooms? (N = 14), no. (%)**						
0–3 mo	3–6 mo	6–12 mo	1–2 y	>2 y	Never	
5 (36)	2 (14)	5 (36)	2 (14)	0	0	
**Did your facility review operating room code-required air change rates as part of your COVID-19 response? (N = 14), no. (%)**						
Yes	No					
11 (79)	3 (21)					
**If you answered yes to the previous question (ie, operating room air-change rates were reviewed because of COVID-19), as a result of the review, what did you do with your air-change rates? (N = 11), no. (%)**						
Increased	No change	Decreased				
2 (18)	8 (73)	1 (9)				
**For a typical operating room at your institution, what percentage of your operating room air exchanges are from outdoor air (rather than from internal return)? (N = 12), no. (%)**						
<20%	20%–24%	25%–29%	30%–35%	35%–99%	100%	
3 (25)	3 (25)	3 (25)	0	2 (17)	1 (8)	
**Did your facility increase the fraction of outside air supplied to operating rooms during COVID-19? N = 14 (%)**						
Yes	No					
4 (29)	10 (71)					
**If you answered yes to the previous question (ie, the fraction of outside air supplied to operating rooms was increased during COVID-19), how was the fraction changed? (N = 1)**						
Updated to current code minimums	Increased as high as the system could go while maintaining comfort	Changed to 100% outside air to potentially reduce the risk of COVID-19 cross contamination				
	1					
**What percentage of your operating rooms use HVAC final filters with the following Minimum Efficiency Reporting Value (MERV) rating? (N = 10), mean %**						
<13	13	14	15–16	17-20 (HEPA filters)		
0	2	0	19	79		
**Prior to COVID-19, how often were operating room HVAC final filters changed (on average with typical use)? (N = 9), no. (%)**						
0–3 mo	3–6 mo	6–12 mo	1–2 y	>2 y		
0	4 (44)	3 (33)	2 (22)	0		
**Did your facility increase the filtration MERV rating of your air handling unit filters during COVID-19? (N = 10), no. (%)**						
Yes	No, not desired	No filters available	No, exceeds HVAC design limitations			
1 (10)	6 (60)	1 (10)	2 (20)			
**Did your facility retrofit the operating room HVAC system to accommodate COVID-19 patients? (N = 10), no. (%)**						
Yes	No					
2 (20)	8 (80)					
**Which of the following (before COVID-19), if any, devices do you use in your operating room to try to prevent surgical site infections? (Check all that apply.) (N = 10), no. (%)**						
UV light robot	Standalone HEPA filters	In duct UV or photocatalytic	Ceiling-based UV	Other (please list)	None	
5 (50)	0	2 (20)	1 (10)	0	4 (40)	
**Which of the following, if any, extra HVAC precautions did you take in your operating rooms during COVID-19? (N = 10), no. (%)**						
UV light robot	Standalone HEPA filters	In duct UV or photocatalytic	Ceiling-based UV	Anteroom retrofit and/or negative pressure peration room	Other (please list)	None
3 (30)	1 (10)	1 (10)	0	4 (40)	1 (10) – “1 negative pressure room)	3 (30)
**In your opinion, in general, national standards for air exchange rates in operating rooms should ______. Please use free text to state why you made your choice. (N = 10), no. (%)**						
Increase	Decrease	Stay the same	I don’t know			
2 (20)	0	6 (60)	2 (20)			
**Does your hospital have an environmental sustainability or green initiative/committee/officer? (N = 10), no. (%)**						
Yes	No	I don’t know				
5 (50)	5 (50)	0				
**How much do you agree/disagree with the following statement:** **“Pollution caused by the healthcare industry is important to minimize.” (N = 9), no. (%)**						
Strongly agree	Agree	Neutral	Disagree	Strongly Disagree		
4 (44)	5 (55)	0	0	0		
**How much do you agree/disagree with the following statement:** **“I am concerned about climate change.” (N = 9), no. (%)**						
Strongly agree	Agree	Neutral	Disagree	Strongly Disagree		
4 (44)	5 (55)	0	0	0		
**How much do you agree/disagree with the following statement:** **“Energy conservation is NOT important.” (N = 10), no. (%)**						
Strongly agree	Agree	Neutral	Disagree	Strongly Disagree		
0	1 (10)	0	6 (60)	3 (30)		
**How much do you agree/disagree with the following statement:** **“There is no limit to the amount of resources we should use to prevent even one patient from developing a hospital-acquired infection.” (N = 10), no. (%)**						
Strongly agree	Agree	Neutral	Disagree	Strongly Disagree		
1 (10)	0	3 (30)	5 (50)	1 (10)		
**What was your standardized infection ratio for 2018 (publically reported data,** https://www.medicare.gov/hospitalcompare/search.html **?#) for colon surgical site infections? (N = 10), mean (SD)**						
1.05 (±0.7)						
**If you are at a Veterans’ Affairs Hospital, what was your colon surgical site infection rate for 2018? (N = 1)**						
1.00						
**My primary institution is (Check all that apply to your primary institution.) (N = 10), no. (%)**						
Community-based	Academic	Ambulatory	Veterans’ Affairs	Critical access hospital	Other	
3 (30)	6 (60)	0	1 (10)	0	0	
**There are ______ acute-care beds at my primary institution. (N = 10), no. (%)**						
0–25	26–250	261–500	501–750	751–1,000	>1,000	0/ambulatory center
0	2 (20)	4 (40)	2 (20)	1 (10)	1 (10)	0
**In which state is your primary institution? (required to understand weather, climate, and energy mixes only)**						
CT, DC, GA, IN, MD, NH, PA, TX, WA						
**My primary job description is ______. (N = 10), no. (%)**						
Infection preventionisty	Hospital epidemiologist	Engineer	Facilities management	Other		
2 (20)	8 (80)	0	0	0		
**I am _____ years old. (N = 10), no. (%)**						
18–29	30–39	40–49	50–59	60–69	>70	
0	2 (20)	1 (10)	5 (50)	1 (10)	1 (10)	
**I have been in practice for ____ years (not including training). (N = 10), no. (%)**						
<5	6–10	11–15	16–20	21–25	26–30	>30
0	1 (10)	2 (20)	2 (20)	2 (20)	2 (20)	1 (10)
**What is your sex? (N = 10), no. (%)**						
Male	Female	Other	Prefer not to answer			
7 (70)	2 (20)	0	1 (10)			
**What is your race? (N = 10), no. (%)**						
White	African American	American Indian or Alaska Native	Asian	Native Hawaiian or Pacific Islander	Other	Prefer not to answer
8 (80)	0	0	0	0	0	2 (20)

### Air change rates

Of 16 respondents, 14 (88%) did not decrease OR air-exchange rates during off hours before the pandemic. Before the COVID-19 pandemic, among these 14 respondents, 86% checked air exchanges more frequently than annually.

The percentages of air change from outdoor air varied. Overall, 1 respondent reported 100% external (outside) air changes (the most energy-intensive strategy); 2 respondents reported 35%–99% external air changes; and 3 respondents reporting each of the following: <20% external air, 20%–24% external air, and 25%–29% external air. Of 10 respondents, 9 (90%) used HEPA final filters in some ORs, and final filters were changed at least every 2 years in the institutions of all respondents (100%). In response to COVID-19, 6 (60%) of 10 did not increase MERV filter rating because it was not desired, 1 (10%) did not because filters were unavailable, 2 (20%) did not because it exceeded HVAC design, and 1 (10%) increased MERV filter rating (this respondent had <100% OR HEPA filter use at baseline).

Regarding opinions on national standards for OR air change rates, 6 (60%) of 10 felt that they should remain unchanged, 2 (20%) had no opinion or did not know, and 2 (20%) thought that they should increase. None felt that rates should decrease. This question did not specify whether respondents should include consideration of the coronavirus disease 2019 (COVID-19) pandemic.

### COVID-19

Among 14 respondents, 11 (79%) reviewed code-required air-change rates because of COVID-19; 8 (73%) made no changes, 18% increased air-change rates, and 9% decreased air-change rates. Among these 14 respondents, 10 did not increase the fraction of outside air supplied to ORs. Of these 10, 2 (20%) were retrofitted OR HVACs to accommodate COVID-19 patients (ie, turned ORs from positive to negative pressure). Table 1 lists further details.

## Discussion

Despite little evidence to support their use from an infection control perspective,^7^ US healthcare institutions use high energy-intensive air-change rates, even exceeding recommended minimums. Reductions in air changes in acute care has the potential for large energy, financial, and carbon savings.^5,8^ Our survey’s respondents indicated that climate change, pollution, and the healthcare industry’s impact thereon were considered important. Despite absence of evidence of benefit, the extent to which HVAC energy resources should be used to prevent infections varied. For example, ∼90% of respondents did not engage in set-back practices in ORs during off hours.

Approximately 80% of respondents reported that they checked OR air-change rates and filters because of COVID-19, some altered air changes or other presumptive infection prevention measures. There was little agreement about whether or how air-change rate standards should be changed. Such uncertainty perhaps resulted from the paucity of evidence for relationships between air-change rates and infections,^7^ guideline unfamiliarity, or both.

### HVAC standards and set-back opportunity

Evidentiary gaps exist on optimal air-change settings for healthcare facilities.^7^ The current standard (ASHRAE 170) recommends a minimum OR rate of 20 air changes per hour including ≥4 outside air changes. Minimum air-change values are required only while the room is occupied. Shutting off OR ventilation systems off hours results in energy or cost savings without exceeding particle-count thresholds.^9^ Yet, our survey response (85% respondents) suggests that facility operators do not decrease air changes during off hours. This finding may reflect unfamiliarity with the lack of evidence on the relationship between OR ventilation rates and SSI prevention.

Future studies should ascertain relationships between air-change rates and SSIs, using the extant natural experiment of institution-to-institution variability to elucidate opportunities for energy, pollution, and cost savings.

### Pandemic response

ASHRAE created an epidemic task force (https://www.ashrae.org/technical-resources/resources) to collect emerging evidence and guidance. Recommendations ultimately followed current ventilation standards (ASHRAE 62.1 and 170) and encouraged additional air filtration when possible.

Numerous institutions tried reducing risks associated with operating on COVID-19 patients. Overalll, 79% of respondents reviewed air-change rates because of COVID-19, and 73% of these made no changes. This finding inplies that prescribed standard flow rates had already been achieved and/or that the system could not be updated. Moreover, 60% responded that mandated air-change rates should remain unchanged. Outside air fractions varied, and 71% made no changes.

Our survey had several limitations. We had low response rates (30% partial response rate, 15% complete), though this rate is similar to that of other surveys (response rates, ∼20%).^10^ Response rates raise these questions: What gaps in air-change rate understanding remain unfilled, and how can this improve?

In conclusion, high air-change rates in ORs are financially costly and have commensurate, possibly unjustified environmental impacts. With uncertain HVAC efficacy on one hand and certain high financial and environmental impacts on the other, we require evidence indicating whether current OR air-change rates influence SSIs. Even with current standards, opportunity exists for off-hour setbacks to reduce energy expenses, to prevent pollution, and to protect public health.
